# Supplementary material for: Pain persists in mice lacking both Substance P and CGRPα signaling
Source: eLife. 2025 Mar 18;13:RP93754. doi: 10.7554/eLife.93754 (PMC11919252; doi:10.7554/eLife.93754)
Supplement: Supplementary file 1. [file elife-93754-supp1.docx]

**Supplementary File 1. Table summarizing all statistical tests used in the manuscript.**

| **Figure** | **Name** | **Variable (units)** | **N** | **Passed Shapiro-Wilk Test for Normality?** | **Test** | **Comparison** | **Statistic** | **P** | **Post hoc analysis** | **Comparison** | **P (adjusted)** |
| --- | --- | --- | --- | --- | --- | --- | --- | --- | --- | --- | --- |
| **1E** | Substance P Release | NKR1 Activation (deltaF/F0) | WT: 12; DKO: 12 |  | 2-WAY RM ANOVA | Interaction | F (2, 44) = 3.718 | 0.0322 | Sidak's multiple comparisons test | Vehicle - WT vs DKO | 0.86 |
|  |  |  |  |  |  | Drug | F (1.569, 34.51) = 78.37 | <0.0001 |  | Capsaicin - WT vs DKO | 0.013 |
|  |  |  |  |  |  | Genotype | F (1, 22) = 5.226 | 0.0322 |  | SP - WT vs DKO | 0.893 |
| **1H** | CGRP Release | CLR Activation (deltaF/F0) | WT: 18; DKO: 18 |  | 2-WAY RM ANOVA | Interaction | F (2, 68) = 5.317 | 0.0072 | Sidak's multiple comparisons test | Vehicle - WT vs DKO | 0.499 |
|  |  |  |  |  |  | Drug | F (1.106, 37.61) = 22.49 | 0.0001 |  | Capsaicin - WT vs DKO | 2E-04 |
|  |  |  |  |  |  | Genotype | F (1, 34) = 0.006702 | 0.9352 |  | CGRP - WT vs DKO | 0.421 |
| **2A** | von Frey | 50% Threshold (log g) | WT: 14; DKO: 14 | Yes (WT: W=0.9238, P=0.2495; DKO: W=0.9521, P=0.5943) | Unpaired t-test | WT vs DKO | t=1.007 | 0.3231 |  | | |
| **2B** | Pinprick | Response (%) | WT: 13; DKO: 11 | No (WT: W=0.813, P=0.0098; DKO: W=0.7538, P=0.0023) | Mann Whitney U Test | WT vs DKO | U=59.5 | 0.5499 |  | | |
| **2C** | Clip | Attending (s) | WT: 7; DKO: 10 | Yes (WT: W=0.8768, P=0.2127; DKO: W=0.9484, P=0.6502) | Unpaired t-test | WT vs DKO | t=0.6762 | 0.5092 |  | | |
| **2D** | Hargreaves | Latency (s) | Low - WT: 12; DKO: 8; High - WT: 15; DKO: 17 |  | 2-WAY ANOVA | Interaction | F (1, 48) = 0.3387 | 0.5633 | Sidak's multiple comparisons test | Low - WT vs DKO | 0.651 |
|  |  |  |  |  |  | Intensity | F (1, 48) = 9.597 | 0.0033 |  | High - WT vs DKO | 0.991 |
|  |  |  |  |  |  | Genotype | F (1, 48) = 0.5373 | 0.4671 |  |  |  |
| **2E** | Hot Plate | Latency (s) | 52.5 - WT: 15; DKO: 10; 55.5 - WT: 9; DKO: 8 |  | 2-WAY ANOVA | Interaction | F (1, 38) = 0.03579 | 0.851 | Sidak's multiple comparisons test | 52.5 - WT vs DKO | 0.987 |
|  |  |  |  |  |  | Intensity | F (1, 38) = 14.27 | 0.0005 |  | 55.5 - WT vs DKO | 0.918 |
|  |  |  |  |  |  | Genotype | F (1, 38) = 0.1417 | 0.7087 |  |  |  |
| **2F** | Acetone | Licking (s) | WT: 12; DKO: 12 | Yes (WT: W=0.9097, P=0.2113; DKO: W=9371, P=0.4616) | | WT vs DKO | t=0.3759 | 0.7106 |  | | |
| **2G** | Dry Ice | Latency (s) | WT: 19; DKO: 13 | No (WT: W=0.9051, P=0.0602; DKO: W=0.7896, P=0.0051) | Mann Whitney U Test | WT vs DKO | U=111 | 0.643 |  | | |
| **2H** | Capsaicin | Licking (s) | WT: 9; DKO: 8 | Yes (WT: W=0.926, P=0.4439; DKO: W=0.9062, P=0.3278) | | WT vs DKO | t=2.041 | 0.0593 |  | | |
| **2I** | AITC | Licking (s) | WT: 12; DKO: 12 | No (WT: W=0.8549, P=0.0422; DKO: W=0.8314, P=0.0218) | | WT vs DKO | U=44 | 0.1135 |  | | |
| **2J** | Acetic Acid | Writhes (#) | WT: 9; DKO: 7 | Yes (WT: W=0.9108, P=0.3214; DKO: W=9211, P=0.4778) | | WT vs DKO | t=0.3757 | 0.7127 |  | | |
| **2K** | Chloroquine | Scratching Bouts (#) | WT: 8; DKO: 11 | No (WT: W=0.9211, P=0.4392; DKO: W=0.7614, P=0.0029) | | | U=39 | 0.7168 |  | | |
| **2-S1** | Capsaicin Fos | Fos puncta / dorsal horn (#) | WT: 5; DKO: 4 | Yes (WT: W=0.9444, P=0.6974; DKO: W=0.8844, P=0.3597) | | | t=0.5736 | 0.5842 |  | | |
| **2-S2** | Conditioned Taste Aversion | Latency (s) | LiCl- WT: 7; DKO: 6; PBS - WT: 5; DKO: 4 |  | 2-WAY ANOVA | Interaction | F (1, 18) = 0.1361 | 0.7165 | Sidak's multiple comparisons test | LiCl | 0.638 |
|  |  |  |  |  |  | Intensity | F (1, 18) = 75.79 | <0.0001 |  | PBS | 0.966 |
|  |  |  |  |  |  | Genotype | F (1, 18) = 0.5413 | 0.4714 |  |  |  |
| **3A** | CFA -- Hargreaves | Latency (s) | WT: 9; DKO: 10 |  | 2-WAY RM ANOVA | Interaction | F (3, 51) = 0.4715 | 0.7035 | Sidak's multiple comparisons test | 0d - WT v DKO | >0.9999 |
|  |  |  |  |  |  | Time | F (2.117, 35.99) = 17.63 | <0.0001 |  | 1d - WT v DKO | 0.996 |
|  |  |  |  |  |  | Genotype | F (1, 17) = 0.8594 | 0.3669 |  | 2d - WT v DKO | 0.334 |
|  |  |  |  |  |  |  |  |  |  | 7d - WT v DKO | 0.734 |
| **3B** | CFA - von Frey | Threshold (log g) | WT: 6; DKO: 6 |  | 2-WAY RM ANOVA | Interaction | F (3, 30) = 0.07568 | 0.9726 | Sidak's multiple comparisons test | 0d - WT v DKO | 0.998 |
|  |  |  |  |  |  | Time | F (3, 30) = 50.74 | <0.0001 |  | 1d - WT v DKO | >0.9999 |
|  |  |  |  |  |  | Genotype | F (1, 10) = 0.02549 | 0.8763 |  | 2d - WT v DKO | >0.9999 |
|  |  |  |  |  |  |  |  |  |  | 7d - WT v DKO | 0.989 |
| **3C** | PGE2 - Hargreaves | Latency (s) | WT: 10; DKO: 10 |  | 2-WAY RM ANOVA | Interaction | F (5, 90) = 2.053 | 0.0787 | Sidak's multiple comparisons test | 0m - WT vs DKO | 0.738 |
|  |  |  |  |  |  | Time | F (5, 90) = 35.65 | 0.0001 |  | 15m - WT vs DKO | >0.9999 |
|  |  |  |  |  |  | Genotype | F (1, 18) = 3.020 | 0.0993 |  | 30m - WT vs DKO | >0.9999 |
|  |  |  |  |  |  |  |  |  |  | 45m - WT vs DKO | 0.765 |
|  |  |  |  |  |  |  |  |  |  | 60m - WT vs DKO | 0.804 |
|  |  |  |  |  |  |  |  |  |  | 120m - WT vs DKO | 0.025 |
| **3D** | PGE2 - von Frey | Threshold (log g) | WT: 6; DKO: 6 |  | 2-WAY RM ANOVA | Interaction | F (4, 40) = 0.3472 | 0.8444 | Sidak's multiple comparisons test | 0m - WT vs DKO | 0.856 |
|  |  |  |  |  |  | Time | F (2.803, 28.03) = 28.64 | <0.0001 |  | 15m - WT vs DKO | 0.977 |
|  |  |  |  |  |  | Genotype | F (1, 10) = 1.475 | 0.2525 |  | 30m - WT vs DKO | 0.999 |
|  |  |  |  |  |  |  |  |  |  | 45m - WT vs DKO | 0.999 |
|  |  |  |  |  |  |  |  |  |  | 60m - WT vs DKO | 0.9 |
|  |  |  |  |  |  |  |  |  |  | 120m - WT vs DKO | 0.025 |
| **3F** | Capsaicin - Oedema | Swelling (norm.) | WT: 9; DKO: 7 | Yes (WT: W=0.9684, P=0.8811; DKO: W=0.8609, P=0.1541) | Unpaired t-test | WT vs DKO | t=0.8876 | 0.3897 |  | | |
| **3G** | Capsaicin - Extravasation | Optical Density (norm.) | WT: 9; DKO: 7 | Yes (WT: W=0.9829, P=0.9776; DKO: W=0.8732, P=0.1981) | Unpaired t-test | WT vs DKO | t=0.2268 | 0.8239 |  | | |
| **3I** | AITC - Oedema | Swelling (norm.) | WT: 8; DKO: 8 | Yes (WT: W=0.9428, P=0.639; DKO: W=0.8994, P=0.2853) | Unpaired t-test | WT vs DKO | t=0.05078 | 0.9602 |  | | |
| **3J** | AITC - Extravasation | Optical Density (norm.) | WT: 8; DKO: 8 | Yes (WT: W=0.9459, P=0.6697; DKO: W=0.9597, P=0.8076) | Unpaired t-test | WT vs DKO | t=0.5681 | 0.579 |  | | |
| **4A** | SNI - Tactile Allodynia | Threshold (log g) | WT: 8; DKO: 8 |  | 2-WAY RM ANOVA | Interaction | F (5, 70) = 1.161 | P=0.3370 | Sidak's multiple comparisons test | 0d - WT v DKO | 0.952 |
|  |  |  |  |  |  | Time | F (5, 70) = 46.15 | P<0.0001 |  | 2d - WT v DKO | 0.731 |
|  |  |  |  |  |  | Genotype | F (1, 14) = 0.01651 | P=0.8996 |  | 5d - WT v DKO | 1 |
|  |  |  |  |  |  |  |  |  |  | 7d - WT v DKO | >0.9999 |
|  |  |  |  |  |  |  |  |  |  | 14d - WT v DKO | 0.753 |
|  |  |  |  |  |  |  |  |  |  | 21d - WT v DKO | 0.991 |
| **4C** | SNI - Fos staining | Fos puncta / dorsal horn (#) | WT: 4; DKO: 3 |  | 2-WAY ANOVA | Interaction | F (1, 5) = 0.02824 | 0.8731 | Sidak's multiple comparisons test | Ipsi - WT vs DKO | 0.815 |
|  |  |  |  |  |  | Side | F (1, 5) = 18.60 | 0.0076 |  | Contra - WT vs DKO | 0.922 |
|  |  |  |  |  |  | Genotype | F (1, 5) = 0.3993 | 0.5552 |  |  |  |
| **4D** | Oxaliplatin - Cold Plate | Behaviors (s) | WT: 9; DKO: 3 | Yes (WT: W=0.9402, P=0.5843; DKO: W=0.9756, P=0.9274) | Unpaired t-test | WT vs DKO | t=0.8884 | 0.3905 |  | | |
